# Fiber Type‐Specific Proteomic Alterations in R349P Desminopathy Mice

**DOI:** 10.1002/mus.28379

**Published:** 2025-03-03

**Authors:** Britta Eggers, Karin Schork, Michael Turewicz, Katalin Barkovits, Martin Eisenacher, Rolf Schröder, Christoph Stephan Clemen, Katrin Marcus

**Affiliations:** ^1^ Medizinisches Proteom‐Center, Medical Faculty Ruhr‐University Bochum Bochum Germany; ^2^ Medical Proteome Analysis, Center for Protein Diagnostics (PRODI) Ruhr‐University Bochum Bochum Germany; ^3^ Core Unit for Bioinformatics (CUBiMed.RUB), Medical Faculty Ruhr‐University Bochum Bochum Germany; ^4^ Institute for Clinical Biochemistry and Pathobiochemistry, German Diabetes Center (DDZ), Leibniz Center for Diabetes Research at the Heinrich Heine University Düsseldorf, Düsseldorf Germany and German Center for Diabetes Research (DZD) München‐Neuherberg Germany; ^5^ Institute of Neuropathology, University Hospital Erlangen Friedrich‐Alexander University Erlangen‐Nürnberg Erlangen Germany; ^6^ German Aerospace Center Institute of Aerospace Medicine Cologne Germany; ^7^ Center for Physiology and Pathophysiology, Institute of Vegetative Physiology, Medical Faculty University of Cologne Cologne Germany

**Keywords:** desminopathy, fiber types, laser microdissection, proteomics, skeletal muscle

## Abstract

**Introduction/Aims:**

Desminopathies are a group of rare human myopathies and cardiomyopathies caused by pathogenic variants of the desmin gene. Here, we analyzed the effects of the R349P mutant desmin on the proteomic profiles of individual fiber types of murine skeletal muscle.

**Methods:**

Soleus and tibialis anterior muscles from hetero‐ and homozygous R349P desmin knock‐in mice and wild‐type siblings were used to collect fiber type‐specific material by laser microdissection to determine their proteomic profiles.

**Results:**

Aberrant proteomic profiles were observed in all four fiber types of homozygous mice. Type I and IIa fibers from homozygous muscle showed an increased abundance of 15 fibrotic proteins, for example, collagens I, IV, and VI, and associated proteins. Filamin‐C, xin actin‐binding repeat‐containing proteins 1 and 2, and Kelch‐like protein 41 were more abundant in homozygous fibers. A high number of proteins associated with the mitochondrial complexes had markedly lower amounts in all types of homozygous and type IIb heterozygous fibers, whereby 20 proteins of complex I, 6 proteins of complex III, 7 proteins of complex IV, and 4 proteins of complex V were found to be decreased in homozygous mice in at least one fiber type. This reduction included all mtDNA‐encoded proteins of complexes I and V, as well as ADP/ATP translocase 1 and 2.

**Discussion:**

Our proteomic findings highlight a more severe myodegenerative process in fibers derived from homozygous R349P desmin knock‐in mice. R349P desmin altered the abundance of proteins of the sarcomeric and extrasarcomeric cytoskeleton, extracellular matrix, and mitochondrial energy metabolism.

**Abbreviations**
DDA: data dependent acquisition
DIA: data independent acquisition
ECM: extracellular matrix
FC: fold change
HCD: high‐energy collision‐induced dissociation
HET: heterozygous
HOM: homozygous
iRT: indexed retention time
LMD: laser microdissection
NCE: normalized collision energy
PCA: principal component analysis
WT: wildtype

## 1 Introduction

The term “desminopathies” refers to a group of rare human myopathies and cardiomyopathies caused by pathogenic variants of the desmin gene (*DES*) on chromosome 2q35 [1, 2, 3]. Since the first description, a multitude of inherited and de novo desmin mutations have been described [2, 4], causing a broad spectrum of familial and sporadic (cardio)myopathies. The majority of desminopathies are autosomal‐dominantly inherited. While the autosomal‐dominant forms are characterized by a co‐expression of wildtype and mutated desmin, the rare autosomal‐recessive forms can be further differentiated into cases with a lack of desmin [5, 6, 7, 8] and cases with sole expression of mutated desmin either without [9] or with [1, 10, 11, 12, 13] protein aggregates.

Desmin is the major intermediate filament protein in muscle cells. In striated muscle, desmin is an essential component of the extrasarcomeric cytoskeleton, which exerts multiple roles in the alignment and anchorage of myofibrils, the positioning of mitochondria and myonuclei, mechanosensation, stress endurance, and cell signaling [2, 14, 15, 16]. Studies on the molecular pathophysiology of desminopathies are hampered by the limited amounts of available human muscle tissue specimens and the fact that alterations noticed in diagnostic muscle biopsies usually reflect late stages of the disease. Thus, cell and animal models that closely mirror the human disease pathology are of paramount importance. We reported on the generation and characterization of hetero‐ and homozygous R349P desmin knock‐in mice that harbor the ortholog of the most frequent human desmin pathogenic variant, R350P. These desminopathy mice develop a desmin‐positive protein aggregation pathology, skeletal muscle weakness, dilated cardiomyopathy, as well as cardiac arrhythmias and conduction defects [17].

In the present work, we investigated the effects of the mixed expression of R349P and wild‐type desmin, as well as the sole expression of R349P desmin, on the proteomic profiles in all skeletal muscle fiber types of the R349P desminopathy mouse model.

## 2 Methods

### 2.1 R349P Desmin Knock‐In Mouse Model

An R349P desmin knock‐in mouse model (B6J.129Sv‐*Des* ^tm1.1Ccrs^/Cscl MGI:5708562) was used, as described in detail [17]. Since previous studies have shown a more pronounced myopathological process in older animals compared to younger mice, the animals utilized in this study were aged 14 to 17 months. The mice were handled in accordance with the German Animal Welfare Act (Tierschutzgesetz) as well as the German Regulation for the protection of animals used for experimental purposes or other scientific purposes (Tierschutz‐Versuchstierverordnung), and the investigations were approved by the responsible governmental animal care and use office (Landesamt für Natur, Umwelt und Verbraucherschutz North Rhine‐Westphalia [LANUV NRW], Recklinghausen, Germany; reference numbers 84‐02.04.2014.A262 and 84‐02.05.40.14.057). Information on sex, genotype, and age can be found in Table 1.

**TABLE 1:** Sample information including sex, genotype, age in months and days.

Mouse ID	Sex	Genotype	Age in months	Age in days
1	F	HOM	17	524
2	F	HOM	17	524
3	M	HOM	17	528
4	M	HET	15	469
5	M	HET	15	469
6	M	HET	15	466
7	M	WT	14	448
8	M	WT	14	448
9	M	WT	14	448

### 2.2 Preparation of Skeletal Muscle Cryosections and Immunostaining of MYH Isoforms

Preparation of skeletal muscle cyrosections was carried out as described [18]. Mice were euthanized, and soleus and tibialis anterior muscles were frozen in isopentane pre‐cooled liquid nitrogen and stored at −80°C. Muscles were cryosectioned into 10 μm thick slices (Cyrostat Microm HM550, Thermo Fisher Scientific, Bremen, Germany), placed on a PET membrane frame slide (Leica, Biosystems, Wetzlar, Germany) for laser microdissection (LMD), and immunostained [18, 19] (Table S1). Antibodies directed against myosin heavy chain (MYH) isoforms were purchased from the Developmental Studies Hybridoma Bank (DSHB), Iowa City, Iowa. For illustration purposes, the immunostaining of soleus muscle sections with antibodies directed against MYH7 and MYH2 is shown (Figure S1).

### 2.3 Sample Preparation for Mass Spectrometry, Mass Spectrometric Measurements, and Data Analysis

Isolation and collection of fiber type material, lysis, tryptic digestion, and mass spectrometric measurements were carried out as described [18]. Detailed information on the methodology can be found in the Supporting Information: Materials and Methods, including the annotation of raw files to samples (Table S2), samples used to create a spectral library (Tables S3 and S4), resulting quantified and normalized data (Table S5), and statistical evaluation of the data (Tables [Link], [Link]).

## 3 Results

### 3.1 The Sole Expression of R349P Desmin Induces Distinct PCA Clusters in All Fiber Types

**FIGURE 1:**
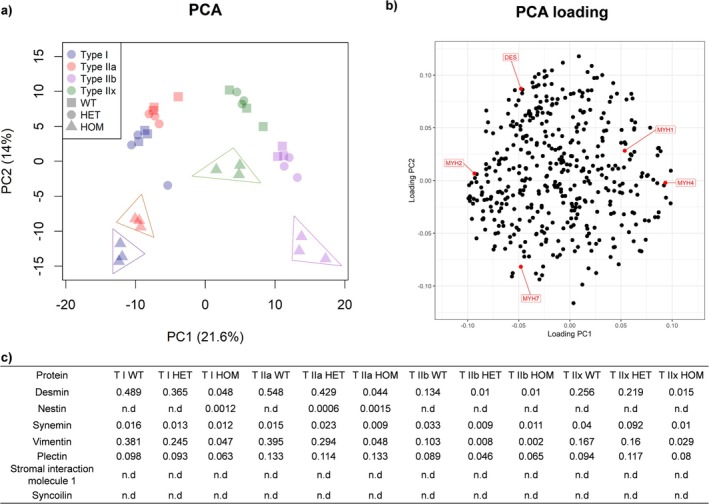
(a) Principal component analysis displayed a segregation of fiber type proteomes based on individual fiber types (type I in blue, type IIa in red, type IIb in purple and type IIx in green) and genotypes (wildtype = WT (square), heterzygous = HET (circle), homozygous = HOM (triangle)). HOM fibers (framed) separately clustered from their WT and HET fiber type counterparts with HOM type I and type IIa fibers forming a combined cluster. This segregation indicates that the sole expression of mutant desmin has a more severe impact than the combined expression of wild‐type and mutant desmin. (b) Corresponding PCA loading indicated that the myosin heavy chain (MYH) isoforms and desmin mainly contributed to the separation into components. (c) Average Percentage aLFQ values of desmin, nestin, plectin, synemin, vimentin, plectin, stromal interaction molecule 1and syncoilin in all fiber types and genotypes. The abundance of desmin was markedly reduced in all fiber types from HOM and type IIb fibers from HET mice. Vimentin displayed a similar abundance pattern. Synemin was found to be lowest in TIIa, TIIb and TIIx HOM and TIIb HET fibers. n.d., not quantified.

We here analyzed the fiber type‐specific protein expression pattern in fibers derived from soleus and tibialis muscles of heterozygous (HET) and homozygous (HOM) R349P desminopathy mice and wildtype (WT) siblings. A principal component analysis (PCA) determined differences or similarities in the global protein pattern of the investigated skeletal muscle fibers, showcasing that the fiber type and the genotype were the two major determinants in this setting (Figure 1a). There were large differences between HOM and both HET and WT fibers for all fiber types. The exclusive expression of the desmin pathogenic variant had the strongest impact on HOM type I and type IIa fibers that resulted in a combined cluster (Figure 1a surrounded in blue and red). In contrast, HET and WT fibers showed similar protein patterns across all fiber types, indicating that the mixed expression of WT and the desmin variant did not cause major changes in the proteomic profiles between these two genotypes. With PCA loading (Figure 1b), we were able to map the strongest separators for all groups. Fiber types mainly differed in their composition of myosin heavy chain (MYH) isoforms (Table S7), as well as their desmin expression abundance.

### 3.2 The Fiber Type‐Specific Expression of Desmin and Desmin Interaction Partners

In a next step, we calculated the absolute abundances of desmin and its direct interaction partners (nestin, synemin, vimentin, plectin, syncoilin, stromal interaction molecule 1) in all fiber types (Figure 1c and Table S12). Highest desmin abundance was found in WT type IIa (0.548%) and WT type I (0.489%) fibers, whereas WT type IIx and WT IIb exhibited lower levels (0.256% and 0.134%, respectively). In contrast, all types of HOM fibers and type IIb HET fibers were almost devoid of desmin (values between 0.01% and 0.048%, Figure 1c). Nestin, which was not detectable in any fiber type of WT mice, was present at very low levels in type IIa HET and type I and IIa HOM fibers. Synemin levels were similar in type I fibers of all three genotypes, higher in type IIa and IIx HET fibers, but lower in type IIa, IIx, and IIb HOM and type IIb HET fibers. Vimentin was markedly reduced in all fiber types of the HOM genotype and type IIb HET fibers, and moderately reduced in type I and IIa but unchanged in type IIx HET fibers. For plectin, present in all fibers of all genotypes, no clear pattern could be determined. The desmin interaction partner syncoilin was not detected in any sample, and stromal interaction molecule 1 could not be quantified since no unique peptides were detected in the mass spectrometric analysis.

### 3.3 Effect of R349P Desmin on Protein Expression in Different Fiber Types

**FIGURE 2:**
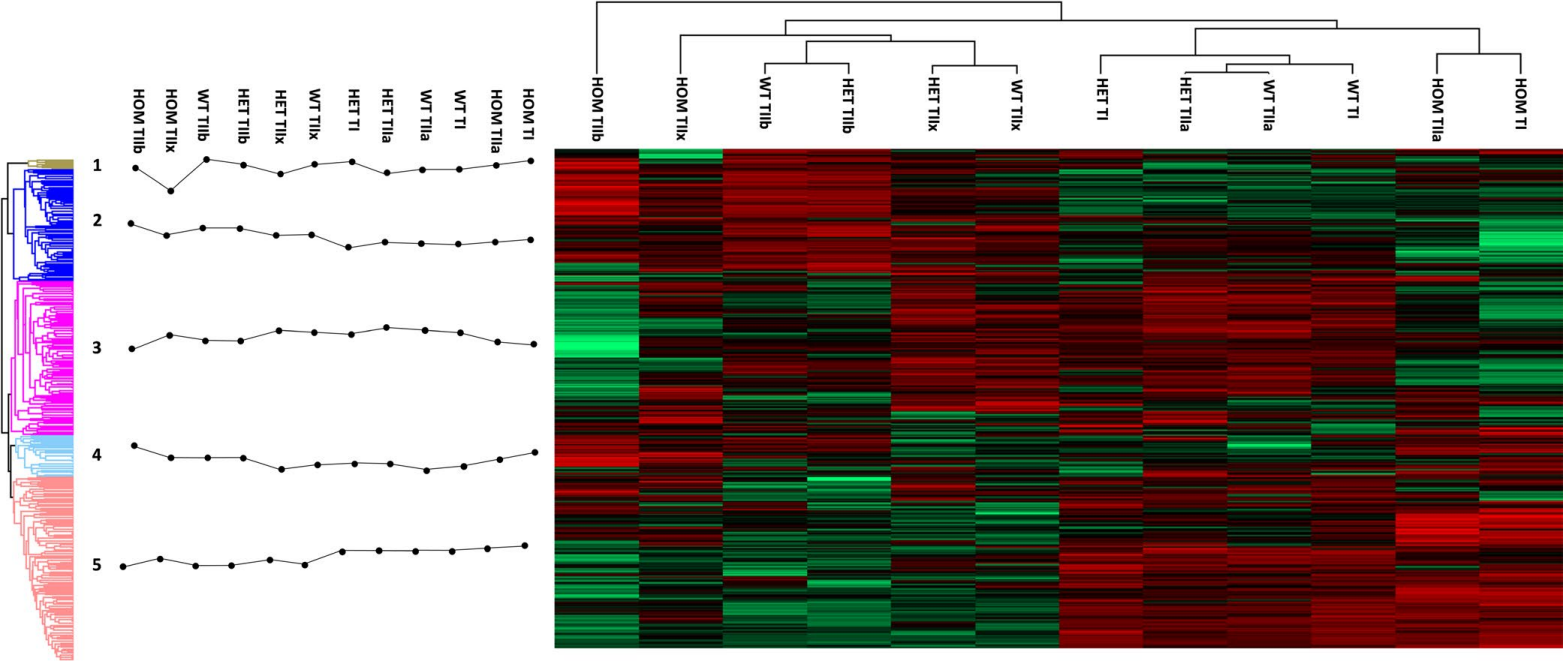
Hierarchical clustering of protein intensities (red = highest intensity, green = lowest intensity). The resulting decision tree (top right), denoted differences in protein expression levels of fibers from the three genotypes. The created heat map was subdivided into 5 clusters (left) with the corresponding profile plot (black dotted lines) visualizing summed intensities for every fiber type (T) and genotype (wildtype = WT, heterozygous = HET, homozygous = HOM). Clusters 2 and 3 referred to proteins associated with energy supply pathways, namely cluster 2 with glycolysis and cluster 3 with oxidative phosphorylation. Clusters 1 and 4 were mainly composed of ribosomal proteins. Cluster 5 was enriched in proteins associated with ECM and muscle architecture and force generation.

We then performed clustering of quantified proteins based on their expression levels (Figure 2, top, decision tree), which again showed marked differences between HOM and WT, but a high degree of similarity between HET and WT for all fiber types. As expected, all fiber types displayed distinct and characteristic protein expression profiles within each genotype. The heat map was divided into five clusters (Figure 2, left side). Clusters were further investigated by GO term and pathway enrichment analysis (Table S10). In keeping with published results [18, 20, 21, 22, 23, 24], clusters 2 (91 proteins) and 3 (127 proteins) were assigned to metabolic pathways involved in energy supply. Cluster 3 was annotated as “respiratory chain and oxidative phosphorylation”, because 15 of 18 GO terms were related to mitochondrial terms, such as “mitochondrial respiratory chain complex III” with enrichment scores over 100‐fold. Key proteins of cluster 2 were involved in the glycolysis (fold enrichment 16‐fold) and the citrate cycle (fold enrichment 38‐fold). Cluster 5 (152 proteins) included an enrichment of GO terms spanning “extracellular matrix (ECM) composition”, such as the dystroglycan‐ (fold enrichment 103 fold) and the sarcoglycan complex (fold enrichment 86 fold) as well as proteasomal processes. Lastly, clusters 1 (8 proteins) and 4 (34 proteins) were mainly associated with the ribosome and the endoplasmic reticulum.

### 3.4 Regulation of Proteins Across Genotypes and Fiber Types

We next compared single protein intensities of all fiber types and genotypes by means of a quantitative comparison (Tables [Link], [Link]). The highest number of differential proteins could be detected in type IIa fiber comparisons between WT/HOM animals, followed by the type I fiber comparison and the type IIb fiber and type IIx fiber comparison between WT/HOM animals. According to our PCA results, all comparisons between WT and HET genotypes regardless of the fiber type only yielded a low number of differentially expressed proteins, indicating a higher similarity at the proteome level. To assess whether the proteins being differential in HOM fibers compared to the WT were identical to those being differential between HET and WT fibers, we conducted an overlap analysis. Indeed, between 31% and 65% of proteins were found to be shared (Figure S2). Hence, we limited the further in‐depth fiber type data analysis to the WT and HOM genotypes.

### 3.5 On the Abundances of Mitochondrial Energy Metabolism‐Related Proteins in Fibers Expressing Solely R349P Desmin

The global proteome analysis revealed marked energy metabolism‐related changes in HOM fibers (Tables [Link], [Link]). Mitochondrial proteins accounted for the largest portion of downregulated proteins (55%) in HOM type I fibers (Table S11). Annotation of all differential proteins with respect to energy supply showed a decreased expression of proteins in HOM type I fibers associated with the respiratory chain, the oxidative phosphorylation, the citrate cycle, as well as the fatty acid beta oxidation. For the respiratory chain, all complexes except complex II were affected (23 proteins, Table S11). Specifically, 15 proteins associated with complex I, 2 proteins of the ETF complex, one protein of complex III, two proteins of complex IV, and three proteins of complex V were found to be of decreased abundance in the HOM animals (Table S11). In addition, eight proteins related to the citrate cycle and six proteins associated with fatty acid beta oxidation were found to be underrepresented in HOM type I fibers (Table S11).

In HOM type IIa fibers, 25 proteins associated with the respiratory chain were decreased (13 proteins of complex I, 2 proteins of the ETF complex, 4 proteins of complex III, 2 proteins of complex IV and 4 proteins of complex V). As seen in type I fibers, proteins of the citrate cycle (11 proteins) and of the fatty beta oxidation (3 proteins) also had a lower abundance in HOM type IIa fibers (Table S11). However, two proteins associated with the respiratory chain had a higher abundance in HOM type IIa fibers (Table S7).

The observed changes in mitochondrial metabolism‐related proteins were not limited to type I and IIa fibers, but were also present in type IIx and type IIb fibers of HOM mice (Table S11). Specifically, HOM type IIb fibers presented lower abundances of 10 respiratory chain‐associated proteins (3 proteins of complex I, 2 proteins of complex III, 4 proteins of complex IV and 1 protein of complex V). In addition, three proteins associated with the citrate cycle were decreased in HOM type IIb fibers. In contrast, HOM type IIx fibers showed decreased expression in only five respiratory chain proteins and one protein of the fatty acid beta oxidation. In type IIx fibers, one protein of the respiratory chain showed an increased abundance (Table S9).

**FIGURE 3:**
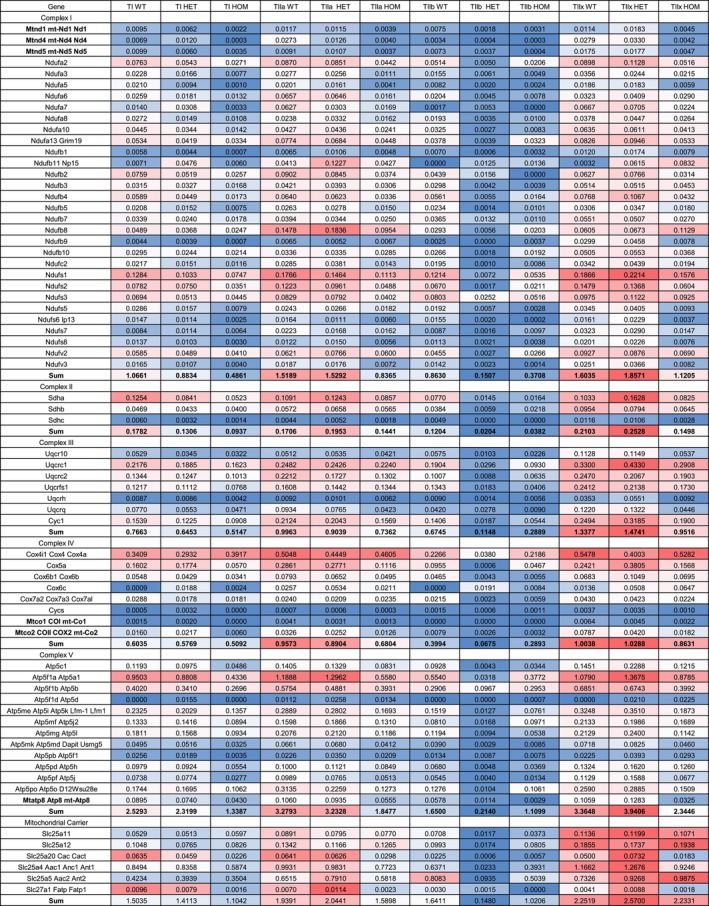
Averaged aLFQ values of identified complex I to V and ETF proteins of the respiratory chain, mitochondrial carrier, and mtDNA‐related proteins for all fiber types (T) and genotypes (wildtype = WT, heterozygous = HET, homozygous = HOM). Color coding of intensities displays highest values in dark red to lowest values in dark blue, within each annotated protein. Mitochondrial (Mt) encoded genes and sum of averaged aLFQ values per complex are marked in bold. HOM fibers displayed the lowest summed protein intensities for all respiratory complexes, except for type IIb fibers that had markedly lower values for each complex in HET animals. The heat map highlighted a pattern in which complex I in type I fibers in HOM animals were predominantly affected.

To obtain more detailed insight into the changes related to the mitochondrial complexes in all fiber types and genotypes, we extended our analysis by counting the total number of quantified proteins of complexes I to V and ETF, and estimated their absolute abundance based on aLFQ values (calculated in %) irrespective of statistical significance (Table S12). Of 50 annotated proteins for complex I, 32 could be quantified in our data set (62%); for complex II, 3 out of 5 proteins (60%); for the ETF complex, one protein; and for complex III, 7 out of 11 annotated proteins were found. For complex IV, only 7 out of 34 annotated proteins could be quantified (21%), and for complex V, 13 (76%) of all 17 proteins could be quantified. Subsequently, we plotted averaged aLFQ intensities for all complexes within the individual fiber types and the three genotypes in a heat map (Figure 3, within each annotated protein highest aLFQ intensities in dark red, lowest in dark blue). HOM fibers displayed the lowest summed protein intensities for all complexes, except for type IIb fibers that had markedly lower values for each complex in HET animals. The heat map highlighted a pattern in which complex I in type I fibers in HOM animals was predominantly affected. Further, it is noteworthy that a ranking of the summed complex intensities unexpectedly did not show the highest values in WT type I and type IIa fibers, but also in HET type IIa and type IIx and HOM type IIx fibers. With regard to mtDNA‐encoded proteins, such as Mtnd1, Mtnd4, Mtnd5, Mtco1, Mtco2, and Mtatp8 that displayed very low intensities as compared to nuclear DNA‐encoded proteins in WT fibers, a trend towards reduced protein levels was also present in all HOM fiber types and HET type IIb fibers.

### 3.6 Impact of R349P Desmin on the Extracellular Matrix, Cytoskeletal Proteins, and Myofibrils

Since our global analysis revealed a dysregulation of ECM, myofibrillar, and other cytoskeletal proteins (Figure 2) we next focused on their quantitation. Across all fiber types from HOM animals, a total of 17 proteins associated with the ECM, 8 cytoskeletal proteins including desmin, and 27 myofibrillar proteins were differentially expressed (Table S11). All regulated ECM proteins were identified as being more abundant in HOM fibers, except for 3 laminin subunits that were downregulated. Out of the 17 regulated ECM proteins, 10 were found to be of higher abundance in HOM type I and 14 proteins in HOM type IIa fibers, among them collagens and collagen‐interacting proteins, for example, prolargin, biglycan, and decorin. In HOM type IIb fibers, only decorin was upregulated, and two laminin subunits were downregulated. In type IIx fibers, only a single protein, laminin subunit beta 1, was regulated.

In the group of cytoskeletal proteins, the R349P desmin was markedly decreased in HOM type I, IIa, and IIx fibers when compared to WT desmin in the corresponding fibers of WT animals. In type I fibers, only one additional cytoskeletal protein was found to be differentially expressed, namely vinculin, which was increased. In HOM type IIa fibers, 4 sarcolemma‐associated cytoskeletal proteins were found to be more abundant: dysferlin, alpha‐sarcoglycan, beta‐sarcoglycan, gamma‐sarcoglycan, and integrin beta‐1. Gamma‐sarcoglycan was also upregulated in type IIb and type IIx fibers, with a marked upregulation of 8.88 in the latter.

Regulated myofibrillar proteins in HOM type I fibers comprised 9 proteins of higher abundance, among them the xin‐acting binding‐repeat‐containing proteins 1 and 2, myosin light chain 6B, kelch‐like protein 41, and the slow fiber type marker myosin‐7. The three regulated fast fiber type markers fast skeletal muscle troponin T, fast type myosin‐binding protein C, and myosin‐2 were of lower abundance in HOM type I fibers. HOM type IIa fibers displayed 11 myofibrillar proteins being of higher and 2 of lower abundance. Again, xin‐acting binding‐repeat‐containing protein 1 and kelch‐like protein 41 were found to be increased, alongside several slow fiber type markers, for example, myosin‐7, myomesin‐1, and the slow fiber type isoform of troponin I, while the fast fiber type marker myosin‐binding protein C2 was decreased. In addition, nebulin was markedly upregulated in the HOM type IIa fibers. HOM type IIb and type IIx fibers only showed upregulated myofibrillar proteins, among them slow fiber type markers alpha‐actinin‐3 and myozenin‐1, but also fast fiber type markers, myomesin‐3 and the developmental myosins 3 and 8. Filamin‐C displayed an increase across the three fiber types I, IIa, and IIb, and kelch‐like protein 41 was found to be the only protein of higher abundance across all HOM fiber types.

## 4 Discussion

In the present study, we used a spatially resolved approach [18] to determine detailed proteomic profiles of all fiber types of soleus and tibialis anterior muscle from R349P desminopathy mice [14, 15, 17, 25, 26, 27]. In keeping with the histopathological analysis of this model, which only demonstrated a clear myopathic phenotype in HOM soleus muscle [17], aberrant proteomic profiles were mainly observed in HOM fibers. This finding again underlines the notion that the mixed expression of WT and R349P desmin leads to desmin protein aggregates, but not to an overt myopathic alteration and muscle weakness in murine skeletal muscle tissue [17].

### 4.1 Levels of Desmin, Desmin Interaction Partners, and Other Extrasarcomeric Cytoskeletal Proteins

Regarding the level of desmin in WT, HET, and HOM fibers, we were able to corroborate previous immunoblot results in which the total amounts of desmin in soleus and extensor digitorum longus muscle tissues were moderately reduced in HET and significantly reduced in HOM animals. Additional R349P desmin‐specific immunoblots showed higher levels in HET than in HOM [14, 17]. This is in line with our previous observation of an R349P desmin‐induced accelerated decay of both WT and variant desmin in HET and HOM skeletal muscle [17]. With the current approach, we added information on desmin abundances on the level of fiber types, whereby the highest desmin abundance was found in WT type IIa and WT type I fibers, while all types of HOM fibers were almost devoid of desmin. The abundances of vimentin followed the same pattern as observed for desmin. Plectin, a giant cytolinker protein with an essential role in the organization and anchorage of the desmin intermediate filament network [28], and the intermediate filament synemin [25] were detected in all fiber types of all genotypes, but no conclusive pattern of differences could be determined. Furthermore, a variety of proteins involved in the attachment of the extrasarcomeric cytoskeleton were found to be either upregulated in HOM type I or type IIa fibers. The overall pattern likely reflects adaptive changes in response to the disorganization of the extrasarcomeric cytoskeleton due to R349P desmin or in response to the subsequent remodeling and increased amount of endomysial connective tissue [2, 26, 27].

### 4.2 R349P Desmin‐Induced Changes in ECM and Myofibrillar Proteins

We detected several regulated ECM proteins in the microdissected muscle fibers, likely reflecting alterations of the ECM microdomains surrounding the specific fiber types. Previous studies already described an increase of endomysial connective tissue in HOM soleus and extensor digitorum longus muscles [14]. As a pattern exclusively related to type I or IIa HOM fibers, the abundances of fibrotic proteins comprising collagens I, IV, and VI, and fibrillin‐1, as well as fiber‐associated proteins like asporin, biglycan, microfibrillar‐associated protein 2, fibulin‐5, decorin, fibromodulin, and thrombospondin‐4 were markedly increased, highlighting the crosstalk between diseased muscle cells and their surrounding connective tissue environment.

The defective desmin intermediate filament network further leads to misalignment, faulty angular orientation, and various degrees of degeneration of myofibrils, thereby leading to an increased turnover of myofibrillar proteins [14, 15, 29], which is also reflected in our new results. Filamin‐C and xin‐acting binding‐repeat‐containing proteins 1 and 2 are established markers for sarcomeric damage [30, 31]. Both markers were also more abundant in the microdissected HOM fibers. In addition, Kelch‐like protein 41, which plays a role in the assembly and maturation of thin fibrils [32], was upregulated in all HOM fibers. The type I fiber predominance observed in the Myosin‐7 immunofluorescence stains of HOM R349P desmin knock‐in muscle tissue is mirrored in increased abundances of other slow fiber type myofibrillar proteins like myosin light chain 6B and slow fiber type isoform of troponin and decreased values for fast fiber type proteins like myosin‐2, myosin‐binding protein C, and fast skeletal muscle troponin T. Along with the expression of the developmental myosins 3 and 8, this pattern reflects an augmented turnover and regeneration process of the myofibrillar apparatus in the diseased fibers.

### 4.3 A Fiber Type‐Specific Look Into the Mitochondrial Pathology in R349P Desminopathy Mice

Building on published work on secondary mitochondriopathy in human R350P desminopathy [29, 33] and the corresponding R349P desmin knock‐in mice [17, 34], our current analysis led to the detection of significantly higher numbers of regulated mtDNA‐and nuclear DNA‐encoded mitochondrial proteins in all fiber types from all genotypes analyzed. In keeping with our previous immunoblotting [17] and proteomic results in HOM soleus muscle tissue [34], mitochondrial proteins accounted for the largest portion of downregulated proteins in all fiber type comparisons. Given the observation that the HOM expression of R349P desmin is associated with multiple large‐scale mtDNA deletions [34], it is noteworthy that all mtDNA‐encoded proteins of complexes I and V showed the lowest expression levels in HOM fibers regardless of the fiber type (Figure 2 and Table S11). In addition, our work confirms the finding in cultured HOM R349P desmin knock‐in myotubes of a reduction of proteins essential for mitochondrial antiport and proton gradient maintenance [35]. ADP/ATP translocase 1 showed a reduction of protein abundance of almost 30% in HOM type I fibers, while in HOM type IIa, type IIb, and IIx fibers, the protein abundance was reduced by 14%, 48%, and 21%, respectively (based on aLFQ). Similarly, abundance values of ADP/ATP translocase 2 were found to be lower in HOM type I, IIa, and IIb fibers. The electrogenic aspartate/glutamate antiporter was found to be mildly reduced in type I fibers by 22%. Taken together, these findings highlight the noxious effect of R349P desmin on mitochondria, which is much more prominent in murine muscle cells that solely express the pathogenic variant.

## Author Contributions

**Britta Eggers:** conceptualization, methodology, investigation, data curation, visualization, formal analysis, writing – review and editing, writing – original draft, validation. **Karin Schork:** methodology, software, visualization, investigation, writing – review and editing. **Michael Turewicz:** methodology, visualization, software, investigation, writing – review and editing. **Katalin Barkovits:** supervision, writing – review and editing, resources. **Martin Eisenacher:** funding acquisition, writing – review and editing, resources, software. **Rolf Schröder:** conceptualization, resources, writing – review and editing, writing – original draft, funding acquisition, supervision, project administration. **Christoph Stephan Clemen:** conceptualization, methodology, supervision, resources, project administration, writing – review and editing, writing – original draft, funding acquisition. **Katrin Marcus:** conceptualization, supervision, resources, project administration, writing – review and editing, funding acquisition.

## Disclosure

We confirm that we have read the Journal's position on issues involved in ethical publication and affirm that this report is consistent with those guidelines.

## Conflicts of Interest

Britta Eggers, Karin Schork, Katalin Barkovits, Martin Eisenacher, and Katrin Marcus are funded by the Ruhr‐University Bochum, Bochum Germany and declare no conflicts of interest. Michael Turewicz is funded by the German Center for Diabetes Research (DZD e.V.), München‐Neuherberg, Germany and declares no conflicts of interest. Rolf Schröder is funded by the University Hospital Erlangen, Friedrich‐Alexander University Erlangen‐Nürnberg, 91054 Erlangen, Germany and is a consultant to MIRA Vision Microscopy GmbH, but declares no conflicts of interest. Christoph Stephan Clemen is funded by the German Aerospace Center, Institute of Aerospace Medicine and the Center for Physiology and Pathophysiology, Institute of Vegetative Physiology, Medical Faculty, University of Cologne. He further is a consultant to MIRA Vision Microscopy GmbH, but declares no conflicts of interest.

## Supporting information


**Table S1.** Information on utilized antibodies for fiber‐type specific stainings.

**Table S2.** Mass spectrometric raw file numbers and the corresponding sample type (containing mouse ID, genotype and fiber type).

**Table S3.** Samples used for Spectral Library generation and corresponding mass spectrometric raw files (*n* = 69). Detailed information on samples utilized to create a spectral library for data independent acquisition. For the generation of a spectral library muscle tissue of wildtype (WT), heterozygous (HET) and homozygous (HOM) mice were pooled and measured in data dependent acquisition mode. Pooled samples were either measured as a complex sample or were fractionated via high pH reverse fractionation prior the management resulting in 8 fractions. Additionally, laser microdissected muscle tissue was analyzed to mimic a sample utilized in our study. For all included samples information on genotype, muscle type and age is provided.

**Table S4.** Spectral library. Spectral library generation was carried out using Spectronaut Pulsar and the Pulsar search engine (Biognosys, Schlieren, Switzerland). Data were searched against the Uniprot KB *Mus musculus* reference proteome set including iRT peptides (53,560 entries) and a contaminant database resulting in a library size of 28,107 peptides and 5005 proteins.

**Table S5.** Normalized protein intensities after LOESS normalization.

**Table S6.** Quantitative comparison of protein intensities of all proteins between wildtype (WT), heterozygous (HET) and homozygous (HOM) type I fibers, providing information on protein accession, protein name, *q* value (protein groups), *p* value (ANOVA), and adjusted *p* values (ANOVA FDR), *p* value (Student’s *t*‐tests), and fold changes (FC) of differential proteins for the respective comparison.

**Table S7.** Quantitative comparison of protein intensities between wildtype (WT), heterozygous (HET) and homozygous (HOM) type IIa fibers, including information on protein accession, protein name, *q* value (protein groups), *p* value (ANOVA) and adjusted p‐values (ANOVA FDR), as well as *p* value (Student’s *t*‐tests) and fold changes (FC), of differential proteins for the respective comparison.

**Table S8.** Quantitative comparison of protein intensities between wildtype (WT), heterozygous (HET) and homozygous (HOM) type IIb fibers, including information on protein accession, protein name, *q* value (protein groups), *p* value (ANOVA) and adjusted *p* values (ANOVA FDR), as well as *p* value (Student’s *t*‐tests) and fold changes (FC), of differential proteins for the respective comparison.

**Table S9.** Quantitative comparison of protein intensities between wildtype (WT), heterozygous (HET) and homozygous (HOM) type IIx fibers, including information on protein accession, protein name, *q* value (protein groups), *p* value (ANOVA) and adjusted *p* values (ANOVA FDR), as well as *p* value (Student’s *t*‐tests) and fold changes (FC), of differential proteins for the respective comparison.

**Table S10.** Gene ontology (GO, based on cellular compartments (CC)) and pathway enrichment (based on KEGG pathways) of the different heat map clusters (Cluster 1–5, see Figure 3) utilizing DAVID (**D**atabase for **A**nnotation, **V**isualization and **I**ntegrated **D**iscovery). Included are the GO or respective KEGG term, the number of associated proteins, p‐value, as well as corrected *p* values (Bonfferoni and Benjamini Hochberg) and fold enrichment scores, indicating the enrichment of a certain term compared to a mouse background proteome.

**Table S11.** Quantitative comparison of mitochondrial proteins and proteins associated with the extracellular matrix between wildtype (WT) and homozygous (HOM) fiber types, including information on gene name, protein accession, and fold changes (FC), of differential proteins for the respective comparison. All proteins were found to be statistically significant (*p* < 0.05).

**Table S12.** Calculated absolute label free quantified (aLFQ) values for all proteins, based on the aLFQ tool. The table contains the peptide output of the SpectronautPulsar search, which is needed to calculate protein aLFQ values. Additionally, the original aLFQ script output, as well as normalized values based on percent for all quantified proteins and averaged percentage values for all fiber types and genotypes are provided. Further, information on the quantified proteins (accession and protein name) are given.

**Figure S1.** Immunofluorescence staining of soleus muscle sections of all genotypes. 10 μm cryosections (10x magnification) of soleus muscle from (a) wildtype (WT), (b) heterozygous (HET) and (c) homozygous (HOM) mice. Type I fibers in red stained with anti‐body BA‐F8 specific for MYH7, type IIa fibers in green stained with antibody SC‐71 specific for MYH2.

**Figure S2.** Venn Diagrams displaying the overlaps of differentially expressed proteins (WT/HET, WT/HOM, HET/HOM) within the different genotype comparisons for type I fibers (a), type IIa fibers (b), type IIb fibers (c) and type IIx fibers (d). For all fiber types proteins of higher abundance in homozygous mice versus heterozygous (HET/HOM) or wildtype (WT/HOM) mice had the highest match.

**Data S1.** Supporting Information.

## Data Availability

The data that support the findings of this study are openly available in PRIDE at https://www.proteomexchange.org/, reference number PXD049207.
