# Supplementary figures and images for: Fiber Type‐Specific Proteomic Alterations in R349P Desminopathy Mice
Source: Muscle Nerve. 2025 Mar 3;71(6):1113–21. doi: 10.1002/mus.28379 (PMC12060636; doi:10.1002/mus.28379)

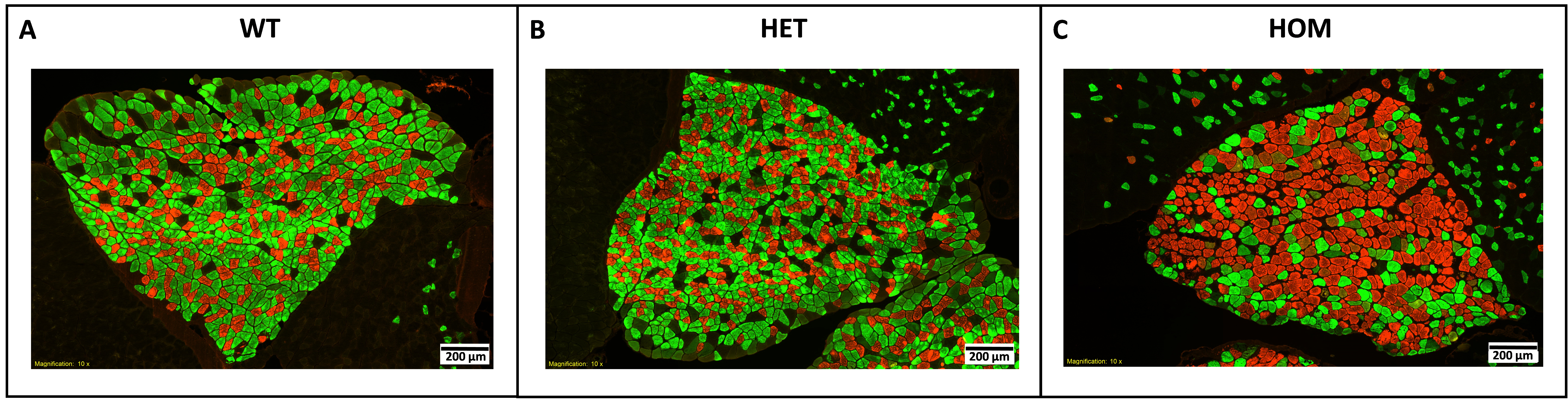

Supplement: Supplementary file 13 — Figure S1. Immunofluorescence staining of soleus muscle sections of all genotypes. 10 μm cryosections (10x magnification) of soleus muscle from (a) wildtype (WT), (b) heterozygous (HET) and (c) homozygous (HOM) mice. Type I fibers in red stained with anti‐body BA‐F8 specific for MYH7, type IIa fibers in green stained with antibody SC‐71 specific for MYH2. [file MUS-71-1113-s010.tif]

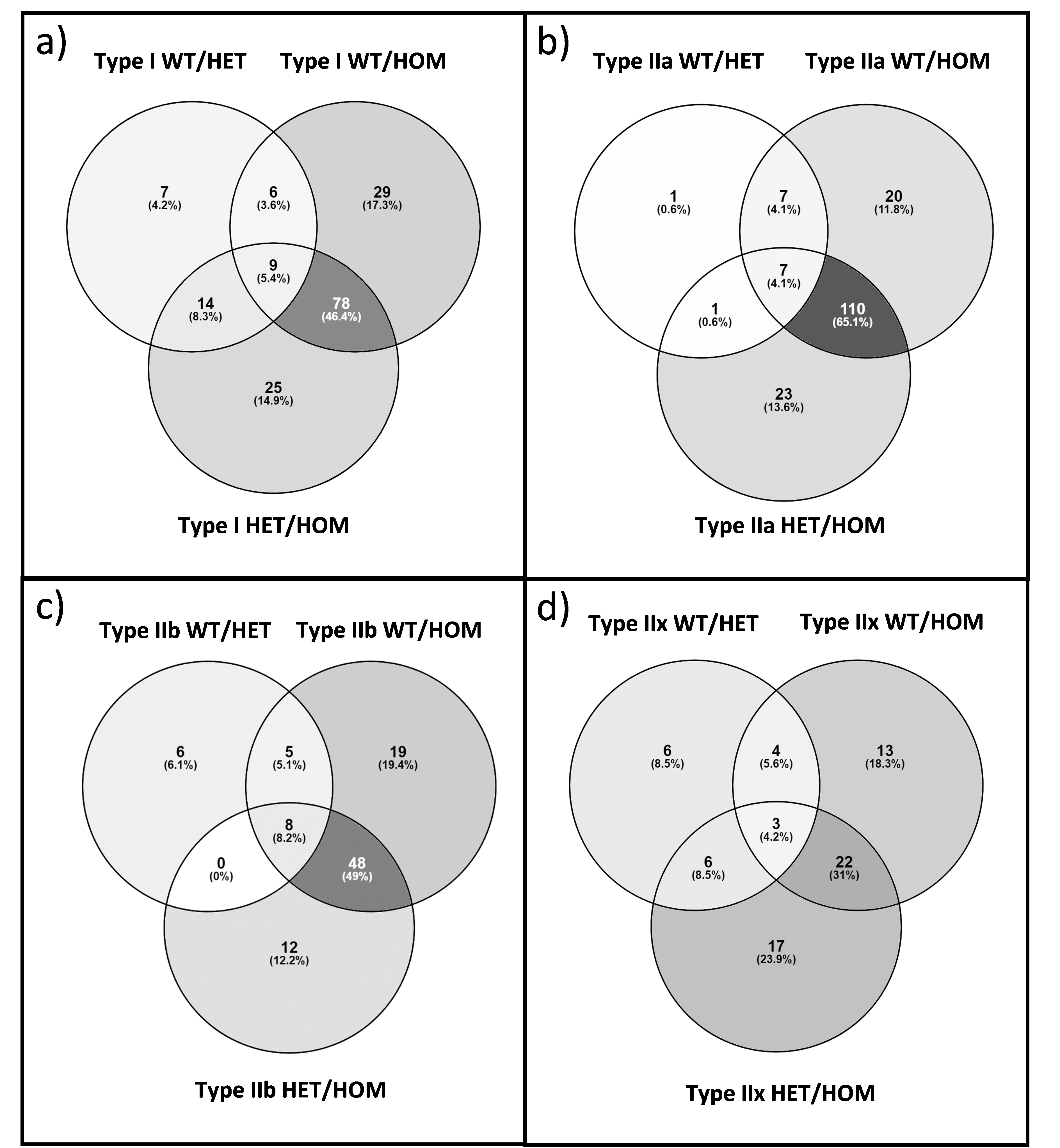

Supplement: Supplementary file 14 — Figure S2. Venn Diagrams displaying the overlaps of differentially expressed proteins (WT/HET, WT/HOM, HET/HOM) within the different genotype comparisons for type I fibers (a), type IIa fibers (b), type IIb fibers (c) and type IIx fibers (d). For all fiber types proteins of higher abundance in homozygous mice versus heterozygous (HET/HOM) or wildtype (WT/HOM) mice had the highest match. [file MUS-71-1113-s011.tif]
