# Supplementary material for: Fiber Type‐Specific Proteomic Alterations in R349P Desminopathy Mice
Source: Muscle Nerve. 2025 Mar 3;71(6):1113–21. doi: 10.1002/mus.28379 (PMC12060636; doi:10.1002/mus.28379)
Supplement: Supplementary file 15 — Data S1. Supporting Information. [file MUS-71-1113-s009.pdf]

Laser microdissection

Laser microdissection (LMD 6500, Leica Microsystems, Wetzlar, Germany) was carried out as described [1,2]. Briefly, 1,000,000 µm² of fibers were excised per each sample and LMD parameters were adjusted for each PET membrane slide. For protein lysis, samples were covered with 40 µL of formic acid and incubated for 20 min at RT, followed by a 5 min sonication step to disrupt tissue and PET membrane. Until further processing samples were stored in -80°C.

Tryptic digestion

Tryptic digestion was carried out as described [2]. Briefly, samples were completely dried in a vacuum concentrator, and 50 µL of 50 mM AMBIC was added as digestion buffer. Reduction and alkylation of cysteine residues was carried out by sample incubation with 15 mM DTT at 56°C for 30 min and subsequent addition of 5 mM IAA and incubation in the dark at RT. To start the digestion, 0.1 µg trypsin was added to each sample. Digestion was carried out over night at 37°C and stopped by acidifying. Samples were again completely dried in the vacuum concentrator and filled up with 50 µL 0.1% TFA, of which 8 µL were taken for mass spectrometric analysis. Additionally, 1 µL of indexed retention time (iRT) peptides (Biognosys) were included in each sample enabling retention time alignment for data independent analysis (DIA).

Lysis of whole soleus and tibialis anterior muscle

For spectral library generation whole murine tibialis anterior and soleus muscles were pulverized in liquid nitrogen and homogenized on ice and afterwards resuspended in urea buffer (7 M urea, 2 M thiourea, 20 mM Trisbase pH 8.5) as described [3]. Resuspended samples were sonicated 6x for 30 sec, with 30 sec rest on ice to support the lysis. Protein concentration was determined by Bradford assay.

High pH reversed phase fractionation

High pH reversed phase fractionation of proteins extracted from whole soleus and tibialis anterior muscle was performed using the PierceHigh pH Reversed-Phase Peptide Fractionation Kit (Thermofisher Scientific, Bremen, Germany) as recommended by the manufacturer. Briefly, 50 µg of digested muscle lysate was loaded on the equilibrated spin column and an increasing organic solvent gradient lead to the fractionation of peptides. The resulting 8 fractions were collected and used for spectral library generation.

Mass spectrometric analysis

Data dependent acquisition (DDA) analysis for spectral library generation was carried out as descried [2]. Briefly, nanoHPLC analysis was performed on an UltiMate 3000 RSLC nano LC system (Thermofisher Scientific). The HPLC system was online-coupled to the nano ESI source of a QExactive HF mass spectrometer (Thermofisher Scientific,). Full MS spectra were scanned in a range between 350 and 1,400 m/z with a resolution of 60,000 at 200 m/z and top ten most intensive ions (charge state +2, +3, +4, AGC target 3e6, 80 ms maximum injection time) were selected for fragmentation experiments and were set on a 30s exclusion list. MS/MS fragments were generated by high-energy collision-induced dissociation (HCD) and the normalized collision energy (NCE) was either set to a fixed value of 27 or a stepped NCE was applied (25.5, 27, 30), enlarging fragment ion spectra annotation. The fragments were analysed in an orbitrap analyser (resolution 30.000, AGC 1e6, maximum injection time 120 ms). Fiber type samples included in the study were analysed via data independent acquisition (DIA, see supplementary table S2). DIA analysis was performed on the same instrument choosing a DIA setting and implementing an inclusion list of specified windows. In total 24 windows were set with a window size of 45 m/z over the complete scan range from 350-1,400

m/z. In the ESI-MS/MS analysis, full MS spectra were scanned in a range from 350 to 1,400 m/z with a resolution of 120,000 at 200 m/z for the detection of precursor ions (AGC target 3e6, 20 ms maximum injection time). MS/MS fragments were generated HCD in which ion dissociation was performed at an NCE of 27%.

Spectral library generation

Data independent acquisition (DIA) [4,5] regularly uses a spectral library for the correct assignment of fragment ion spectra to their respective precursor ion. To generate an optimal DIA method, sample specific libraries were created for all three genotypes using laser microdissected muscle fibers as well as whole soleus and tibialis tissue samples, resulting in a total of 69 DDA runs (for annotated raw files see supplementary table S3). Spectral library generation was carried out using Spectronaut Pulsar and the Pulsar search engine (Biognosys). Data were searched against the Uniprot KB [6] *mus musculus* reference proteome set including iRT peptides (53,560 entries) and a contaminant database resulting in a library size of 28,107 peptides and 5,005 proteins. Biognosys factory settings were applied and trypsin was chosen as digestion enzyme. Specific modifications were set according to sample treatment: carbamidomethylation (C) (fixed modification) and oxidation of (M), deamidation (NQ) and carbamidomethylation (N-term) (variable modifications) were included (for spectral library see supplementary table S4).

Data analysis

DIA analysis war carried out using Spectronaut Pulsar as described [2]. Resulting non normalized LFQ data was used for further data analysis using R Version 3.5.1 (R Core Team, 2018). Non normalized LFQ data were exported and a locally weighted scatter plot smoothing normalization (LOESS) was applied using the limma R package version 3.36.5 [7] (see supplementary table S5). The internal normalization of Spectronaut, as well as median and quantile normalization were also investigated, but an evaluation of MA-Plots showed that

LOESS-normalization was suited best for the data. The data was log2 transformed and to find differentially expressed proteins between the distinct fiber types, a repeated measures ANOVA (R package nlme version 3.1-137) was calculated in all genotypes for each fiber type separately. ANOVA-p-values were corrected using the Benjamini-Hochberg procedure. Proteins with fewer than two non-missing values per group were excluded before the ANOVA (see supplementary tables S6-S9).

Hierarchical clustering and GO term and Pathway enrichment

Non-normalized data obtained from Spectronaut was normalized as described above and LFQ data was used for hierarchical clustering with Perseus [8]. For that, the LFQ data was log transformed. Prior clustering, the data set was filtered by setting a minimum of 50% valid values in total. Existing missing values were replaced from normal distribution by imputing using a width of 0.3 and a downshift of 1.8. Groups were averaged using the median and data was Z-scored prior clustering. For hierarchical clustering Euclidian distance was chosen with an average linkage and no constraint. Preprocessing was carried out as factory settings recommended. K-means was enabled and a value of 300 clusters was chosen with a maximal number of 10 iterations. Pathway and Gene annotation enrichment analysis were carried out using DAVID Bioinformatics Resources 6.8 [9,10]. For that, the functional annotation tool was chosen. Uniprot accession of proteins were uploaded. Here only the first accession for protein group results was taken to enable accurate analysis. Uniprot ID identifier was set and list type was set to gene list. As background the whole *mus musculus* genome was chosen. KEGG pathway was used for the determination of enriched pathways in the dataset. Cellular Compartment was chosen for GO term enrichment studies. Fold enrichment scores as well as FDR were taken as additional options. As threshold factory settings were maintained. Resulting tables were exported into Excel and sorted after p-value corrected values or fold enrichment scores (see supplementary table S10).

Absolute protein concentration estimation using aLFQ

After exporting the non-normalized protein and peptide intensities out of Spectronaut, a locally weighted scatter plot smoothing normalization (LOESS) was applied using the limma R package version 3.36.5 [7,11]. Calculation of protein quantities was carried out using the aLFQ package (version 1.3.5 [12]) within R (version 3.6.1), as recently described in [2]. For each protein an iBAQ value [13] was calculated from the LOESS-normalized peptide intensities and absolute protein quantities were obtained for each sample separately, to ensure a total normalized protein intensity of 200 ng in each sample (see supplementary table S11). Resulting concentrations were calculated as percent and average aLFQ values were determined for each fiber type and genotype.

REFERENCES

1. Maerkens A, Kley RA, Olivé M, Theis V, van der Ven PFM, Reimann J, et al. Differential proteomic analysis of abnormal intramyoplasmic aggregates in desminopathy. Journal of proteomics 2013; 90:14–27. doi:10.1016/j.jprot.2013.04.026.
2. Eggers B, Schork K, Turewicz M, Barkovits K, Eisenacher M, Schröder R, et al. Advanced Fiber Type-Specific Protein Profiles Derived from Adult Murine Skeletal Muscle. Proteomes 2021; 9(2):28. doi:10.3390/proteomes9020028.
3. Oertzen-Hagemann V, Kirmse M, Eggers B, Pfeiffer K, Marcus K, Marées M de, et al. Effects of 12 Weeks of Hypertrophy Resistance Exercise Training Combined with Collagen Peptide Supplementation on the Skeletal Muscle Proteome in Recreationally Active Men. Nutrients 2019; 11(5). doi:10.3390/nu11051072.
4. Bruderer R, Bernhardt OM, Gandhi T, Miladinović SM, Cheng L-Y, Messner S, et al. Extending the limits of quantitative proteome profiling with data-independent acquisition and application to acetaminophen-treated three-dimensional liver microtissues. Molecular & cellular proteomics MCP 2015; 14(5):1400–10. doi:10.1074/mcp.M114.044305.
5. Bruderer R, Sondermann J, Tsou C-C, Barrantes-Freer A, Stadelmann C, Nesvizhskii AI, et al. New targeted approaches for the quantification of data-independent acquisition mass spectrometry. Proteomics 2017; 17(9). doi:10.1002/pmic.201700021.
6. UniProt: the universal protein knowledgebase. Nucleic acids research 2017; 45(D1):D158-D169. doi:10.1093/nar/gkw1099.
7. Ritchie ME, Phipson B, Di Wu, Hu Y, Law CW, Shi W, et al. limma powers differential expression analyses for RNA-sequencing and microarray studies. Nucleic acids research 2015; 43(7):e47. doi:10.1093/nar/gkv007.
8. Tyanova S, Temu T, Sinitcyn P, Carlson A, Hein MY, Geiger T, et al. The Perseus computational platform for comprehensive analysis of (prote)omics data. Nature methods 2016; 13(9):731–40. doi:10.1038/nmeth.3901.
9. Huang DW, Sherman BT, Tan Q, Collins JR, Alvord WG, Roayaei J, et al. The DAVID Gene Functional Classification Tool: a novel biological module-centric algorithm to functionally analyze large gene lists. Genome biology 2007; 8(9):R183. doi:10.1186/gb-2007-8-9-r183.
10. Huang DW, Sherman BT, Lempicki RA. Systematic and integrative analysis of large gene lists using DAVID bioinformatics resources. Nature protocols 2009; 4(1):44–57. doi:10.1038/nprot.2008.211.
11. Bolstad BM, Irizarry RA, Astrand M, Speed TP. A comparison of normalization methods for high density oligonucleotide array data based on variance and bias. Bioinformatics (Oxford, England) 2003; 19(2):185–93.
12. Rosenberger G, Ludwig C, Röst HL, Aebersold R, Malmström L. aLFQ: an R-package for estimating absolute protein quantities from label-free LC-MS/MS proteomics data. Bioinformatics (Oxford, England) 2014; 30(17):2511–13. doi:10.1093/bioinformatics/btu200.

13. Schwanhäusser B, Busse D, Li N, Dittmar G, Schuchhardt J, Wolf J, et al. Global quantification of mammalian gene expression control. Nature 2011; 473(7347):337–42. doi:10.1038/nature10098.
